# Determination of Phenols Isomers in Water by Novel Nanosilica/Polydimethylsiloxane-Coated Stirring Bar Combined with High Performance Liquid Chromatography-Fourier Transform Infrared Spectroscopy

**DOI:** 10.1038/s41598-017-09050-2

**Published:** 2017-08-18

**Authors:** Bei Zheng, Wentao Li, Lin Liu, Xin Wang, Chen Chen, Zhiyong Yu, Hongyan Li

**Affiliations:** 1 Key Laboratory of Drinking Water Science and Technology, Chinese Academy of Sciences, Beijing, 100085 China; 2 Shenzhen Institute of Information Technology, Shenzhen, 518172 China; 3 Northern Engineering Design and Research International Co, Shijiazhuang, 050011 China

## Abstract

A novel nanosilica/polydimethylsiloxane (SiO_2_/PDMS) coated stirring bar was adopted in the sorption extraction (SBSE) of phenols in water, and the high performance liquid chromatography-fourier transform infrared spectroscopy (HPLC-FTIR) was subsequently used to determination of phenol concentration. The SiO_2_/PDMS coating was prepared by sol-gel method and characterized with respect to morphology and specific surface area. The results of field-emission scanning electron microscope (FE-SEM) and N_2_ adsorption-desorption as well as phenol adsorption experiments denoted that SiO_2_/PDMS has larger surface area and better adsorption capacity than commercial PDMS. The extraction efficiency of phenol with SiO_2_/PDMS coated stirring bar was optimized in terms of ion strength, flow rate of phenol-involved influent, type of desorption solvent and desorption time. More than 75% of phenol desorption efficiency could be kept even after 50 cycles of extraction, indicating the high stability of the SiO_2_/PDMS coated stirring bar. Approximately 0.16 mg/L 2, 5-dimethylphenol (2, 5-DMP), which was 34-fold more toxic than phenol, was detected in water through HPLC-FTIR. However, 2, 5-DMP could be oxidized to 5-methy-2-hydroxy benzaldehyde after disinfection in drinking water treatment process. Therefore, the proposed method of SiO_2_/PDMS-SBSE-HPLC-FTIR is successfully applied in the analysis of phenols isomers in aqueous environment.

## Introduction

Phenols, typically toxic organic compounds, have been increasingly released into the aqueous environment due to human activities of industrial and agricultural production, such as pesticide, pharmacy, coking and oil refining, which finally result in the contamination of soil and water environment^1, 2^. Different phenols levels have been detected in the natural rivers^3, 4^. For example, chlorinated phenols have been extensively existed in the surface water and underground water, sediments and soils, and atmosphere^5, 6^. The presence of chlorine atoms can inhabit the activity of benzene ring lyase to prevent the degradation of chlorinated phenols in the ecosystem^7^. Due to the high toxicity and persistence of phenols, the US environmental protection agency (USEPA) lists 129 priority pollutants containing four chlorophenols, and European Union (EU) sets a maximum concentration of 0.5 ug/L for total phenols and 0.1 ug/L for their individual (i.e., PhOH, 2-CP, 2, 4-DCP, 2, 4, 6-TCP, PCP, 2, 4-DMP) concentration in drinking water^8^. The cresols were 5-fold more toxic than phenol, whereas 2-ethylphenol and 3, 4-dimethylphenol (3, 4-DMP) were 11-fold more toxic, and 2, 5- dimethylphenol (2, 5-DMP) was 34-fold more toxic than phenol^9^, indicating that the structure of phenols directly affect their toxicity. Therefore, the determination of phenols in the environment, especially for phenols isomers, is significantly demanded given to their threat on the global environment and the quality of many living species. Monitoring the presence and their quantity of phenols in the environment is a prudent decision on what to look for, when, where, how and why. The task is complicated by spatial and temporal variations in the amount of phenols present in the environment.

Various techniques have been developed to concentrate the trace organic contaminants from water. To deal with the enormous sampling requirement, the passive sampling technique is attracting more and more attention because of its operational simplicity and cost effectiveness. Several passive sampling methods have been reported to extract various contaminants from water, such as semi-permeable membrane devices (SPMD), solid-phase micro-extraction (SPME), and diffusion-gradient in thin-films^10–12^. The SPMDs is the most widely used method for aquatic hydrophobic pollutants enrichment, such as polychlorinated biphenyls (PCBs), polycyclic aromatic hydrocarbons (PAHs) and organochlorine pesticides^13–15^. But SPMDs are limited into the enrichment of non-polar hydrophobic pollutants; in the procedure of sampling, the low flow rate (0.2 m/s) is required and easily barraged and polluted by organisms^16^. To overcome the above shortcomings mentioned above, the stirring bar sorptive extraction (SBSE) is introduced to the passive sampling devices. High sensitivity, good reproducibility, high adsorption capacity, and high recovery of SBSE endow its great potential in environmental organic contaminants concentration analysis^17^. The commercial polydimethylsiloxane (PDMS) coated stirring bar has been used to extract phenols in water, but it usually provides lower extraction capacity because of the intensive polarity of phenols^18, 19^. It is necessary to develop novel SBSE coatings with better extraction capacity for phenols.

Nano-SiO_2_ microsphere (nano-SiO_2_) is a non-toxic and non-contaminated material, which has been widely used as catalytic supporters, bio-pharmaceutical and electronics. Its high surface area, small particle size, good dispersity and large amount of hydroxyl are preferred in the extraction of phenols from water^20, 21^. The purpose of this study is to prepare a novel nano-SiO_2_/PDMS coating and to develop a new method of nano-SiO_2_/PDMS-SBSE coupled with HPLC-FTIR for the determination of phenols isomers in water. The parameters affecting the extraction capacity of phenols by nano-SiO_2_/PDMS-SBSE were investigated, including adsorption time, flow rate, ion strength, desorption solvent and time, and the analytical performance of the proposed method (SiO_2_/PDMS-SBSE-HPLC-FTIR) was evaluated. Finally, the developed method was applied to determine phenols isomers in water, and its possible utilization in water treatment plants was also examined.

## Results and Discussion

### Characterization of PDMS coating and SiO_2_/PDMS coating

**Figure 1:**
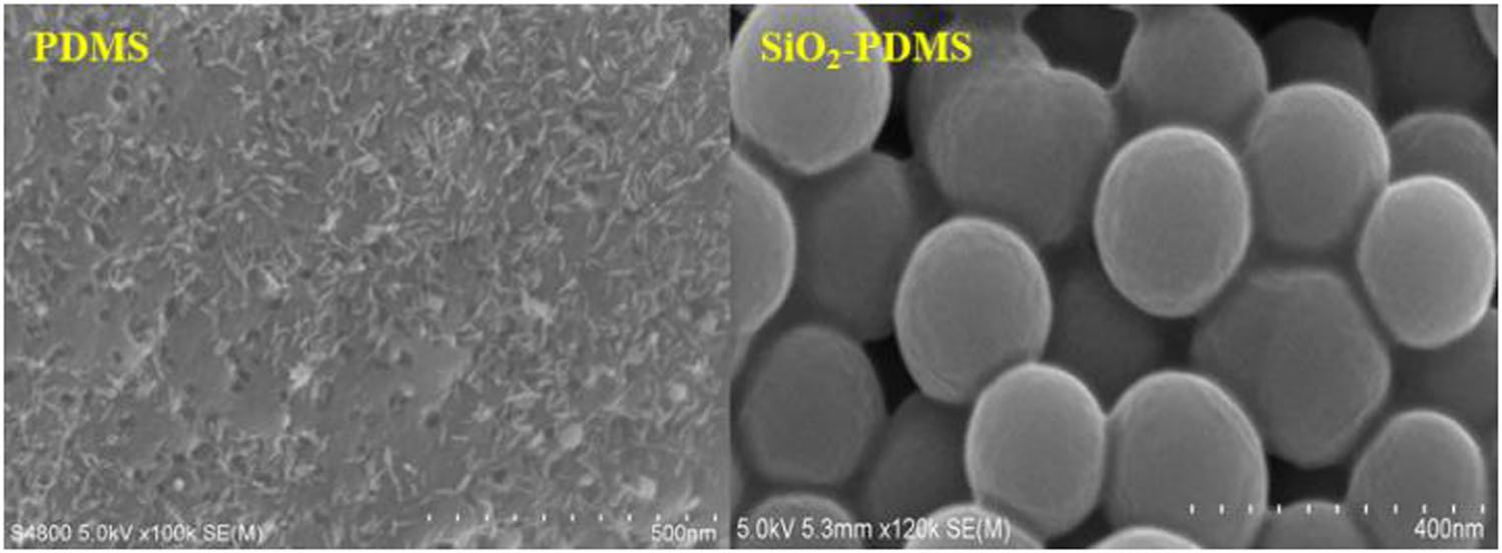
FE-SEM images of PDMS (left) and SiO_2_-PDMS (right).

The morphology of PDMS coating and SiO_2_/PDMS coating was investigated by FE-SEM under the magnification of 100 k and 120 k, respectively, as shown in Fig. 1. Different from the net-like surface of PDMS coating, the surface of SiO_2_/PDMS coating was packed by globular structure, possibly leading to an increase of the surface area. Figure S1 (see in supporting information) shows the IR spectra of SiO_2_/PDMS in the 600–4000 cm^−1^ spectral region. The bands located at 2962 cm^−1^ and 2905 cm^−1^ are unambiguously assigned to symmetrical stretching and asymmetric stretching vibrations of CH_3_. The high intensity band at 1258 cm^−1^ is assigned to the Si-CH_3_ group^22^.The band lying at 1078 cm^−1^ corresponds to Si-O stretching mode. The shoulder that it is observed at 1018 cm^−1^ is due to cyclic structures of PDMS molecules^22^. The peaks at 844 cm^−1^ and 796 cm^−1^ attribute to SiOH-PDMS copolymerization and asymmetric stretching vibration of Si-C^23, 24^.

**Figure 2:**
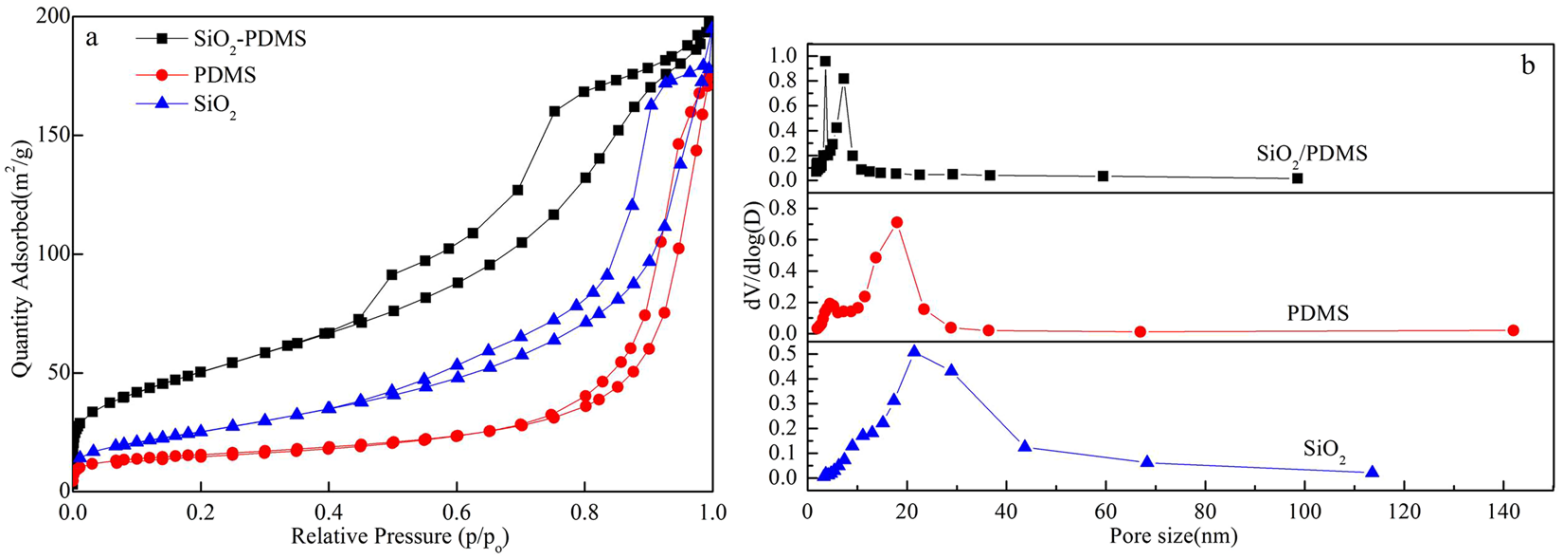
N_2_ adsorption-desorption isotherms (**a**) and pore size distribution (**b**) of PDMS, SiO_2_ and SiO_2_-PDMS.

The Brunauer-Emmett-Teller (BET) characterization of SiO_2_ and SiO_2_/PDMS is given in Fig. 2. The isotherms were found to belong to a typical type IV with type H3 hysteresis loops, indicating that the composite is a typical mesoporous structure with aggregates of plate-like particles. The surface areas and pore size of PDMS, SiO_2_, and SiO_2_/PDMS were 94.12 m^2^/g, 52.50 m^2^/g, 183.69 m^2^/g and 12.82 nm, 20.60 nm, 6.65 nm, respectively, which were listed in Table 1. These data suggest that adsorption efficiency of SiO_2_/PDMS coating can be improved because of its larger specific surface area and smaller pore size.

**Table 1:** Surface area and pore size of samples.

Samples	Surface area(m^2^/g)	Pore volume(cm^3^/g)	Pore size(nm)
PDMS	94.12	0.30	12.82
SiO_2_	52.50	0.27	20.60
SiO_2_-PDMS	183.69	0.31	6.65

### Optimization of SiO_2_/PDMS coating extraction conditions

#### Effects of adsorption time and PhOH initial concentration

**Figure 3:**
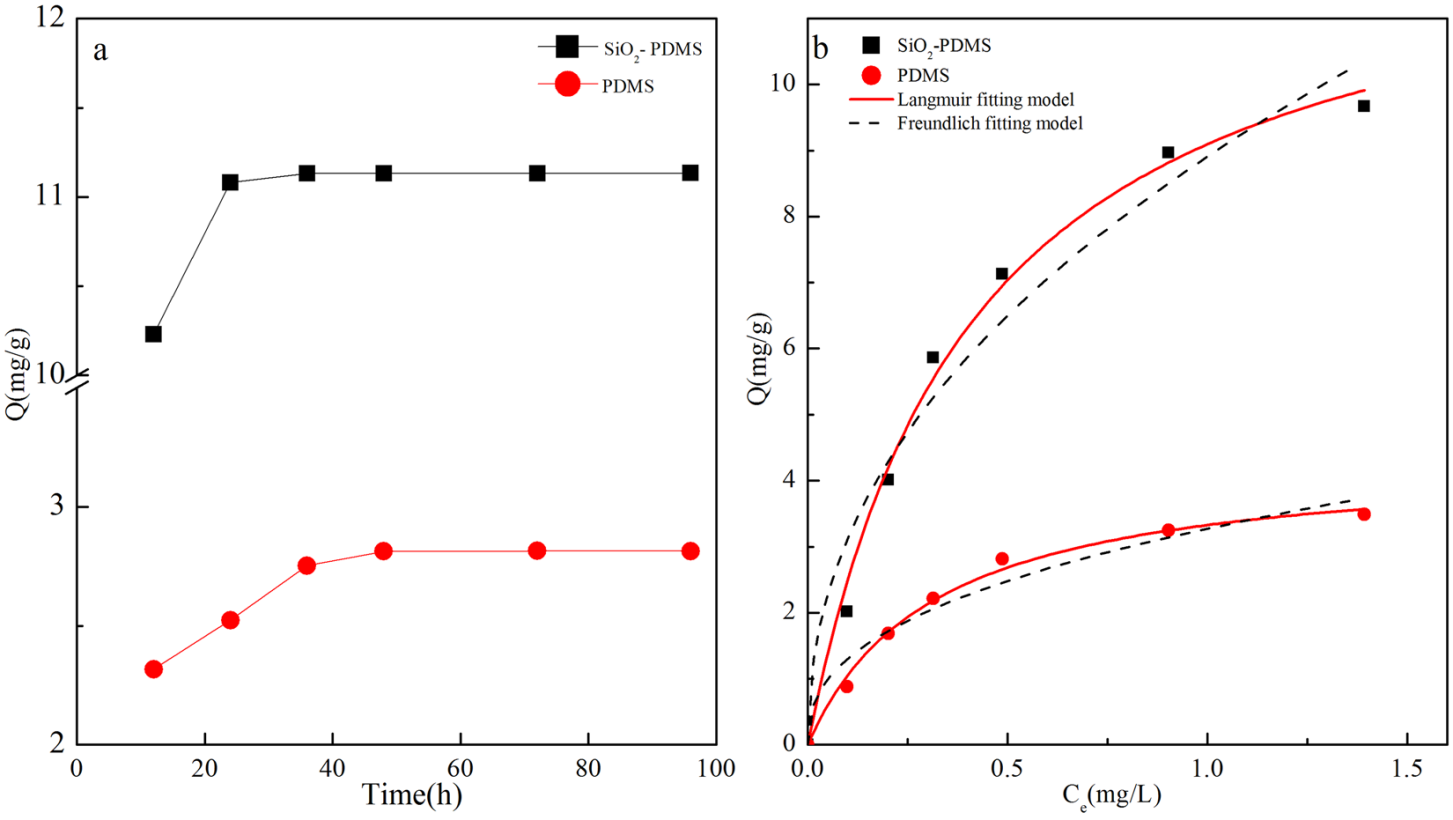
PhOH adsorption kinetics (**a**) and isotherms (**b**) on PDMS or SiO_2_/PDMS.

The adsorption performance of SiO_2_/PDMS coating and PDMS coating was investigated to optimize the adsorption time and the initial PhOH concentration. Figure 3a shows the change of PhOH adsorbing on the SiO_2_/PDMS coating and PDMS coating with adsorption time. The maximum PhOH adsorption capacity on PDMS coating (2.81 mg/g) was found at 48 h, while 11.13 mg/g PhOH adsorption on SiO_2_/PDMS coating was achieved within 24 h. Figure 3b shows the effect of initial PhOH concentration on the uptake of PhOH on SiO_2_/PDMS coating and PDMS coating. The Langmuir and Freundlich isotherm models were used to describe the relationship between the amount of phenol adsorbed and its equilibrium concentration in solutions. All the fitting parameters are listed in Table 2. PhOH adsorption capacity on SiO_2_/PDMS coating is around 3-fold more than that on PDMS coating. Compared to Freundlich equation, the Langmuir equation is better to describe PhOH adsorption behavior, indicating that PhOH adsorption belongs to monolayer adsorption and is irreversible^25^. Therefore, we will choose stronger polar solvent to desorb the PhOH molecules.

**Table 2:** Langmuir and Freundlich constants for PhOH adsorption on PDMS or SiO_2_/PDMS.

Coating types	Langmuir			Freundlich		
	q_m_ (mg/g)	*K*_d_	R^2^	K_f_	n	R^2^
SiO_2_/PDMS	12.87	2.41	0.993	8.91	2.20	0.956
PDMS	4.38	3.17	0.993	3.27	2.50	0.951

Notes: q_m_(mg/g) presents max adsorption amount per unit mass; K_d_, K_f_ and n mean physical parameters in two adsorption isotherm models.

#### Effects of ion strength and flow rate

**Figure 4:**
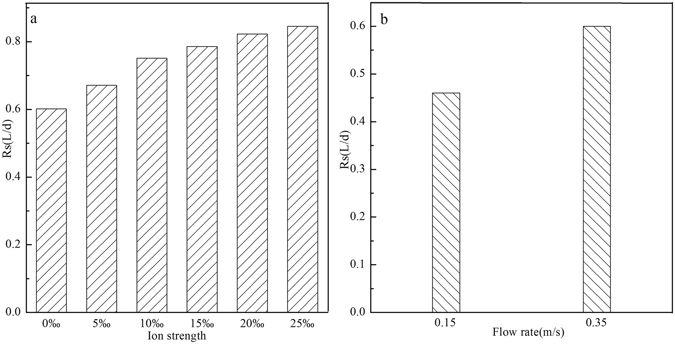
Effects of ion strength (**a**) and flow rate (**b**) on the sampling rate.

The effects of ion strength and water flow rate on the sampling rate are worthwhile to study, which may importantly influence the sampling rate and the analysis results (Fig. 4). In Fig. 4a, the sampling rate for PhOH increased from 0.6 L/d to 0.85 L/d with the increase of ion strength from 0‰ to 25‰. It could be attributed to the decrease of the solubility of polar organic compounds with the increase of ion strength; the insoluble PhOH was then be adsorbed, leading to the higher adsorption rate^26^. Figure 4b shows the influence of flow rate of phenol-involved influent on the sampling rate. When water flow rate increased from 0.15 m/s to 0.35 m/s, the sampling rate for PhOH grew from 0.46 L/d to 0.6 L/d. The water flow rate can significantly change the diffusion layer thickness, and further affect the adsorption kinetic process of target contaminants on the passive sampling devices^27^. In other words, the diffusion layer thickness was reduced with the increasing flow rate, consequently resulting in the increase of contacting opportunities between target contaminants and passive sampling devices^28^.

#### Effects of desorption solvent and time on the PhOH desorption

**Figure 5:**
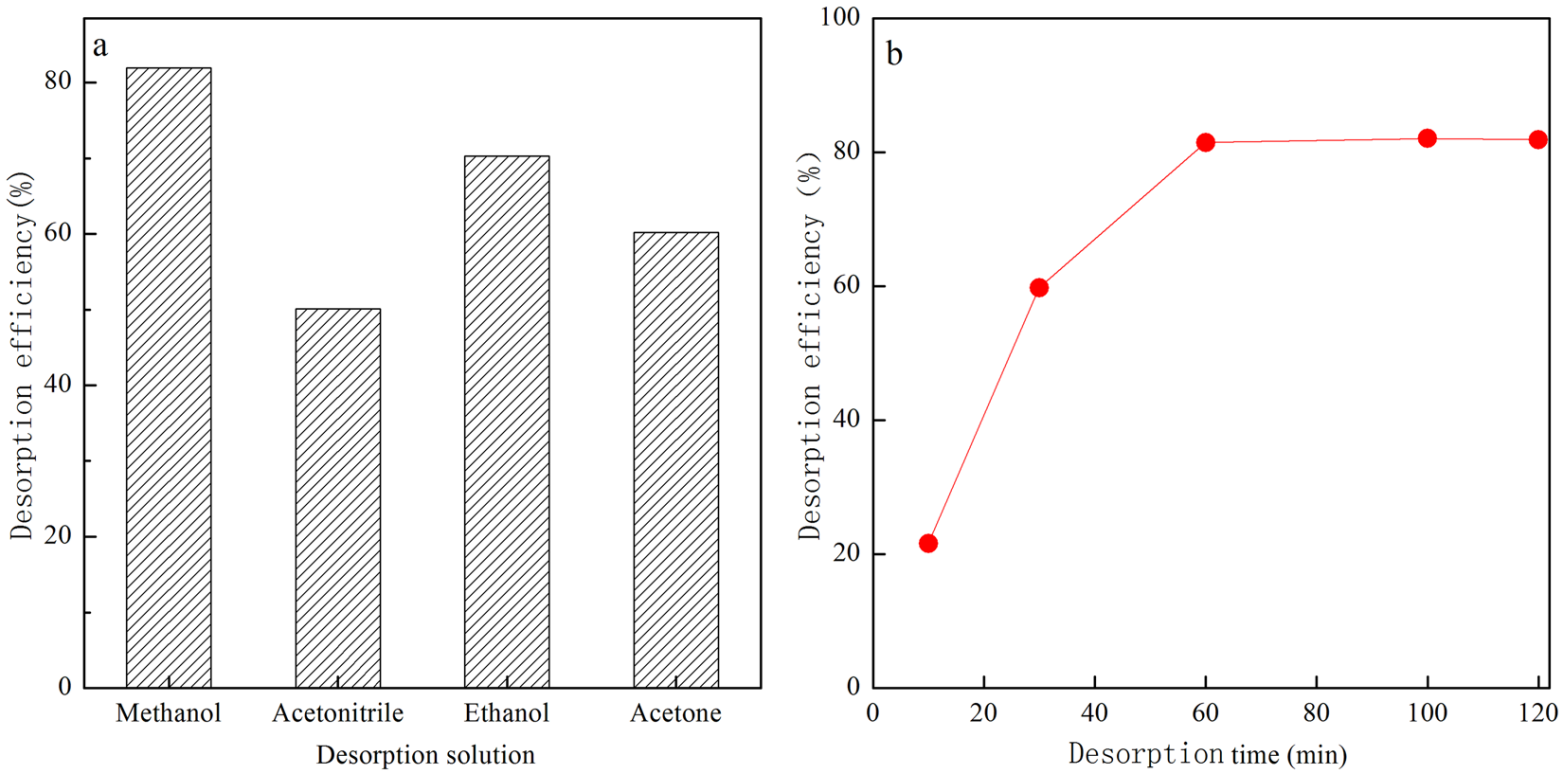
Effects of desorption solvents (**a**) and desorption time (**b**) on the PhOH desorption.

The effect of desorption solvents on desorption efficiency was investigated, as shown in Fig. 5a. Methanol, acetonitrile, ethanol and acetone were used to desorb PhOH from SiO_2_/PDMS coating stirring bar. It was found that PhOH desorption efficiency of methanol, ethanol, acetone, and acetonitrile was 80%, 70%, 60%, 50%, respectively, indicating that methanol provided the best desorption efficiency for PhOH among them. Therefore, methanol was selected as the best optimal desorption solvent for PhOHs^16^. In Fig. 5b, the effect of desorption time ranging from 0 to 120 min on PhOH desorption efficiency from SiO_2_/PDMS coating stirring bar was investigated with methanol as desorption solvent. It can be seen that the desorption equilibrium was achieved at 60 min, which was determined as the optimum desorption time.

#### Life time of SiO_2_/PDMS coating stirring bar

The life time of SiO_2_/PDMS coating stirring bar was evaluated by the desorption efficiency (η), which was calculated by the following equation: η = desorption amount/initial adsorption amount, where initial adsorption means first adsorption. The desorption efficiency was decreased from 86.4% to 78.9% with the increase of cycle frequency (Figure S2), which indicated that there was no obvious loss in the performance of SiO_2_/PDMS coating sorptive stirring bar after 50 rounds of adsorption and desorption cycles. That means the SiO_2_/PDMS sorptive stirring bar can be reused for at least 50 times. Compared with poly (vinylpyridine-ethylene dimethacrylate) and poly (vinylpyrrolididone- divinylbenzene) monolithic coated stirring bars prepared by Huang *et al*.^29, 30^, SiO_2_/PDMS sorptive stirring bar possessed longer life time and shorter adsorption- desorption time.

#### Determination of DMP Isomers Concentration by HPLC-FTIR

The couple of ATR-FTIR and HPLC was investigated for separation and determination of target contaminants^31^, especially for the identification of contaminants isomers that makes up the shortcoming of UV detector.

**Figure 6:**
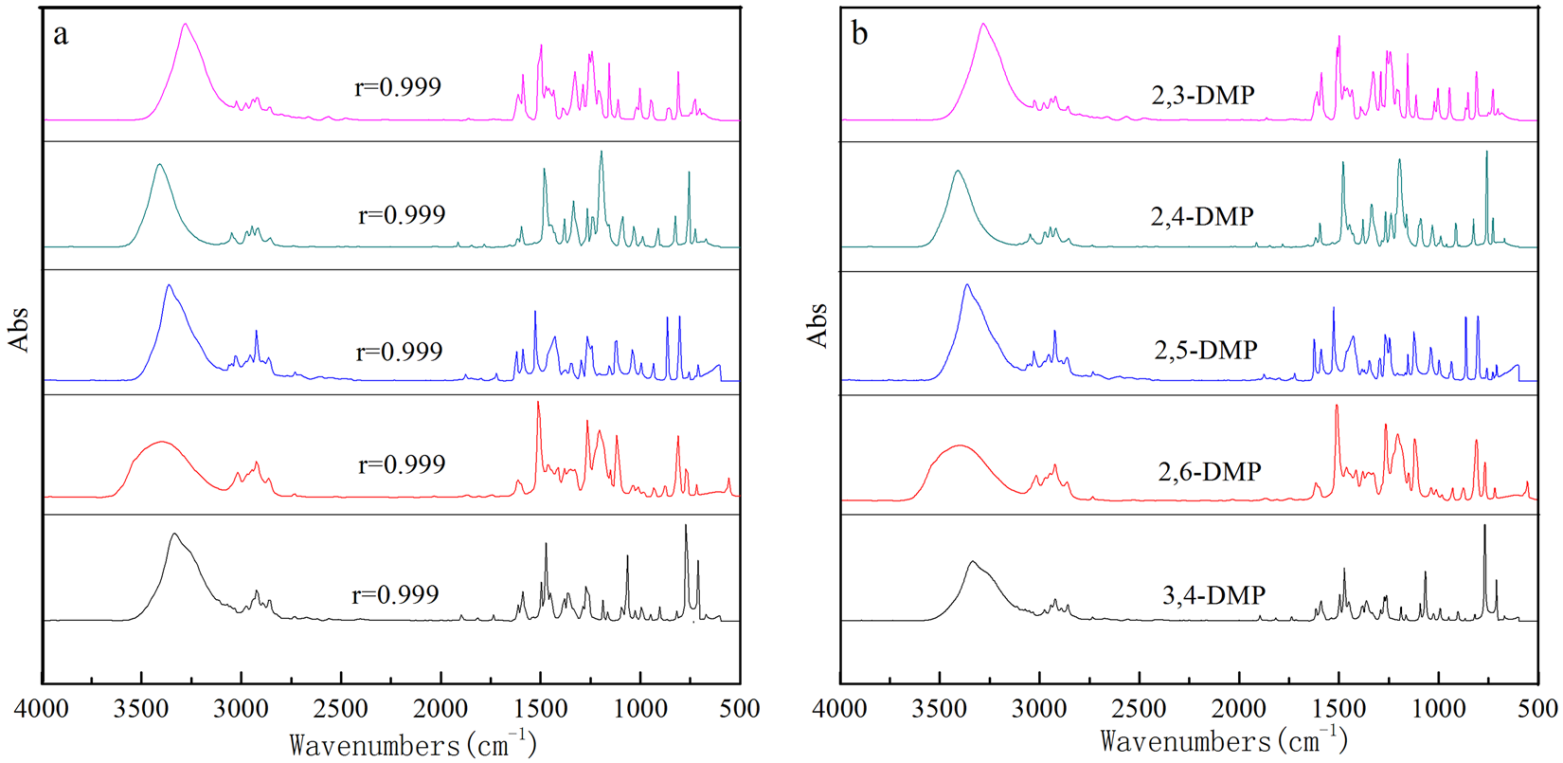
IR spectra of DMP isomers (2,3-DMP, 2,4-DMP, 2,5-DMP, 2,6-DMP, 3,4-DMP): (**a**) IR spectra obtained; (**b**) IR standard spectra.

DMP, as one of the environmental contaminants, has strong potential threats to human health, mainly coming from energy products. And the toxicity of DMP depends on the structure of DMP. Therefore, the determination of DMP isomers is quite significant. But only total phenol amounts were obtained by the traditional ways such as spectrophotometry and fluorescence^32^. To determine phenols isomers, we developed a new method to measure DMP isomers via HPLC-FTIR. Figure 6a shows IR spectra of 1 ug/L DMP could be obtained by HPLC-FTIR. Compared with IR standard spectra of DMP (Fig. 6b), their correlation coefficients were up to 0.999. However, HPLC connected with UV detector could only obtain one chromatographic peak for all DMP isomers at the same retention time (see in Figure S3). This indicates that HPLC-FTIR with fingerprint identification is much more useful to analysis the isomers like DMP.

### Environmental samples analysis

**Figure 7:**
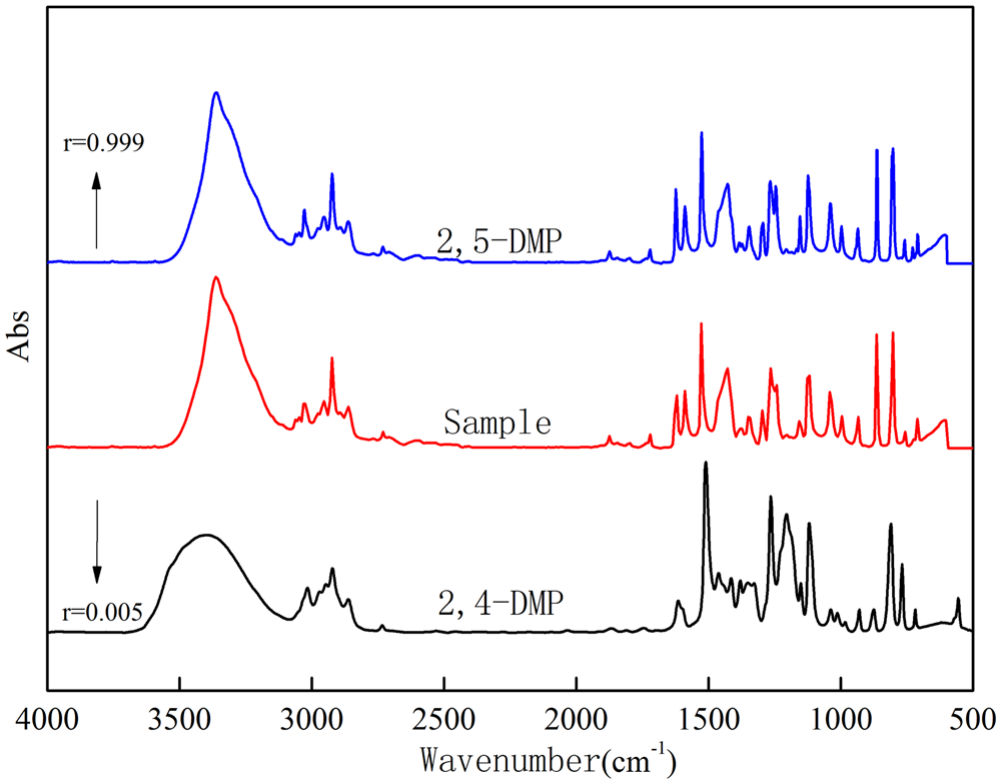
IR spectra of 2, 5-DMP, 2, 4-DMP and sample obtained through HPLC-FTIR in raw water.

Environmental water samples from six water systems were comparatively analyzed via HPLC-FTIR and HPLC-UV. First, water samples were enriched by SiO_2_/PDMS coated stirring bar sorptive extraction, and then these samples were analyzed by HPLC-FTIR. Results showed that 2, 5-DMP was detected instead of 2, 4-DMP through HPLC-FTIR (Fig. 7). The structure of DMP isomer obtained via HPLC-FTIR is quite important to monitor DMP in environment, because different DMP isomer presents different toxicity, as Acuna-Arguelles reported that 2, 5-DMP was 3-fold more toxic than 3, 4-DMP ^9^. However, phenols were not detected in the five water basins, but 0.16 mg/L DMP was detected in another water system through HPLC-UV, which was consistent with results detected by HPLC-FTIR. This suggests that SiO_2_/PDMS- SBSE-HPLC-FTIR technique can be very useful to determine the isomers of phenols contaminants in the environment.

### Application of SiO_2_/PDMS-SBSE-HPLC-FTIR technique in drinking water treatment process

The safety of drinking water treatment plant was further investigated using SiO_2_/PDMS-SBSE-HPLC-FTIR technique. Conventional water treatment process includes coagulation, sediment, filtration and disinfection (see in Figure S4). The reaction conditions are following: 20 mg/L of PAC as coagulant, 10 min of coagulation time and stirring with 30 s^−1^; 1.0 h of sediment time in the flat flow sedimentary pond; activated carbon (particle size, 0.5–1.2 mm; filtering layer thickness, 30 cm) as filter material; 20 mg/L of NaClO as disinfectant and controlling less than 5 mg/L of residual chlorine.

**Figure 8:**
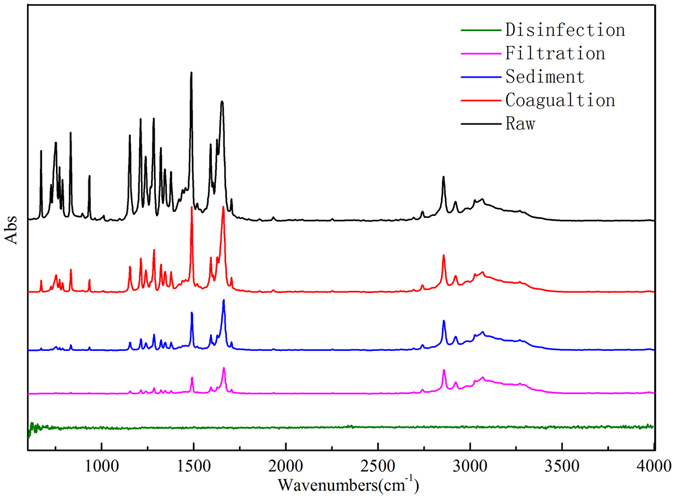
IR spectra of 2, 5-DMP in drinking water treatment process.

After four steps processing, samples were collected and analyzed. In Fig. 8, it can be seen clearly that IR adsorption intensity is obviously decreased through different treatment units, indicating that 2, 5-DMP concentration is reduced. IR signals could be detected after coagulation, sediment and filtration processes, but there is no signal after disinfection (Fig. 8). This may result from 2, 5-DMP oxidation by NaClO to other compounds with aldehyde (equation (1)), such as 3-methyl-4-hydroxy benzaldehyde or 5-methy-2-hydroxy benzaldehyde. By GC-MS analysis, oxidation product is quite similar with the structure of hydroxyl benzaldehyde compounds with one methyl (see in Figure S5), which belongs to isomers of intermediate oxidation.1

**Figure 9:**
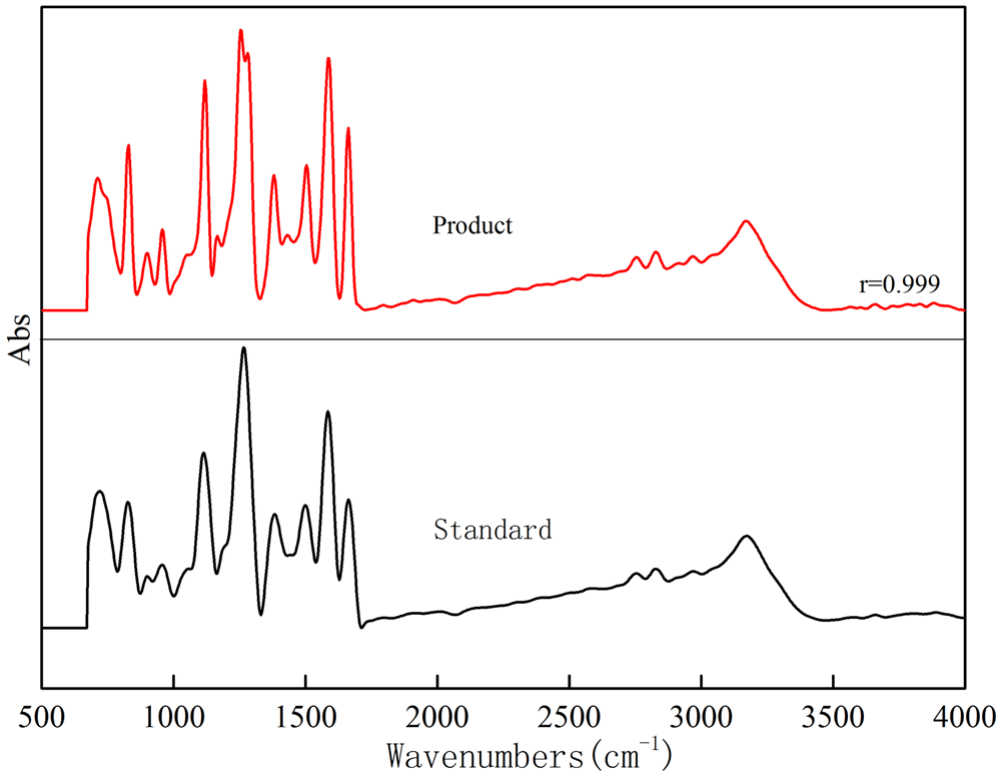
Comparison of IR spectra of disinfection product and standard 5-methy-2-hydroxy benzaldehyde.

To distinguish the isomers’ structure, HPLC-FTIR technique was adopted to identify the molecular structure of disinfection product. The results in Fig. 9 displayed that IR spectrum of disinfection product is completely agreed with that of 5-methy-2-hydroxy benzaldehyde. However, no signal like 5-methy-2-hydroxy benzaldehyde appears in the IR spectra after coagulation, sediment and filtration processes (see in Figure S6). This suggests that 5-methy-2-hydroxy benzaldehyde was only formed in the disinfection process rather than other drinking water treatment processes.

### Experimental section

#### Chemicals

2,4,6-trichlorophenol(2,4,6-TCP, >99.9%), 2-chlorophenol (2-CP, >99.9%), 4-chloro- 3-hydroxytoluene (4-CMC, >99.9%), phenol (PhOH, >99.9%), 2,4-dimethylphenol (2,4-DMP, >99.9%), 2,3-dimethylphenol(2,3-DMP, >99.9%), 2,5-dimethylphenol (2,5- DMP, >99.9%), 2,6-dimethylphenol (2,6-DMP, >99.9%), 3,4-dimethylphenol (3,4- DMP, >99.9%) were purchased from Tokyo Kasei Kogyo Co., Ltd. Potassium bromide(KBr, chromatographically pure) and nano-SiO_2_ (SiO_2_, analytically pure) were purchased from sinopharm chemical reagent co., ltd. Methanol (CH_3_OH, PR) was obtained from thermo fisher scientific. Methytrimethoxysilane (MTMS), polymethylhydrosiloxane (PMHS), methyl silicone resin (MSR), polydimethylsiloxane (PDMS) were purchased from WD silicone co., ltd. Trifluoroacetic acid (TFA), dichloromethane (CH_2_Cl_2_) were obtained from sinopharm chemical reagent co., ltd.

### Preparation of SiO_2_/PDMS coated stirring bar

A 50 mm glass bar with a 41 mm iron core inside was prepared. After cleaned by ultrapure water and CH_2_Cl_2_, respectively, the glass bar was dipped into 1 mol/L NaOH solution for 12 h to expose the silanol groups on its surface. Sequentially, the treated bar was put into 1 mol/L HCl solution for 12 h. Finally, it was cleaned to neutral surroundings and then dried under N_2_ atmosphere at 393 K–423 K.

SiO_2_/PDMS coating was prepared by sol-gel method^33, 34^, as following: 20 mg nano-SiO_2_ was dispersed into CH_2_Cl_2_ containing 200 μL MTMS, 100 μL PMHS, 20 mg PDMS and 100 ul MSR. After mixing uniformly by ultrasonic for 60 min, 0.5 ml TFA was put to the above suspending liquid and then mixed with vortex, producing the gray- white colloid. Sequentially, the colloid was put in the vacuum drying oven for 12 h under room temperature, to remove bubbles in the liquid.

Finally, the treated glass bar was immersed into the mould with the colloid, and then was successively heated at the autoclave for 8 h at 393 K, for 8 h at 473 K, for 8 h at 513 K. Prior to use, SiO_2_/PDMS coated stirring bar was cleaned with methanol.

### Stirring bar extraction experiments

**Figure 10:**
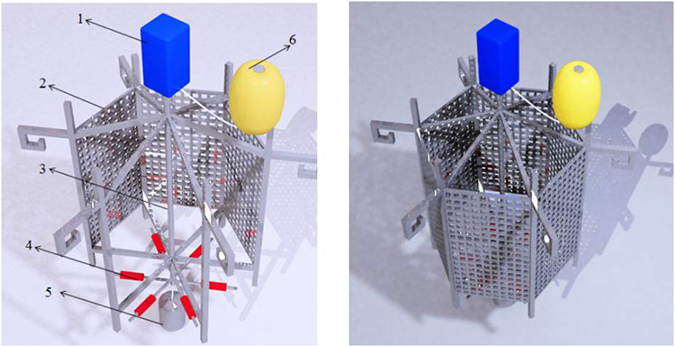
Passive sampler with a sorptive stir bar extraction (SBSE):1, speed control system; 2, metal cage; 3, driving rod; 4, stir bar;5, lead ore;6, buoy.

To compare the extraction property of SiO_2_/PDMS coated with that of commercial PDMS coated, passive sampling device with stirring bar (Fig. 10) was put into the circulating tank to do some experiments on PhOH extraction. In Fig. 10, speed control system connected to driving rod, on the bottom of which six adsorption stir bars were fixed. A metal cage was around them to prevent being damaged from large particles and fishes. In addition, a buoy and lead ore were fixed onto the top and bottom of cage, respectively. By the combination of buoy and lead ore, the passive sampler was suspended onto the water. Under the speed rate, the efficient component loading onto the stir bar could fully contact the contaminants to enrich.

In the experiments, 200 mg NaN_3_ was added to inhibit microorganism growth. The factors that can affect the extraction capacity were investigated, including adsorption time and PhOH initial concentration, ion strength and flow rate, desorption solvent and desorption time.

#### Adsorption tests

0.6 mg/L of PhOH initial concentration was added to the circulating tank. At 0.35 m/s of flow rate, samples were extracted at an interval 12 h from 0 to 100 h. In addition, different PhOH initial concentrations (0.1–1.5 mg/L) were also performed at a flow rate of 0.35 m/s. After 48 h, samples were drawn out. The effects of ion strength (0–25‰) and flow rate of phenol-involved influent (0.15 m/s, 0.35 m/s) on stirring bar extraction were performed with 1 mg/L PhOH initial concentration. These samples were analyzed by HPLC-UV. Sampling rate was calculated in equation (2)^35^:

2 $${\rm{Rs}}=\frac{{\rm{Ms}}}{{{\rm{C}}}_{{\rm{w}}}{\rm{t}}}$$

In equation (2), Rs means sampling rate (L/d), Ms means the target contaminant adsorption at desired time (mg), and C_w_ means target contaminant concentration (mg/L).

#### Desorption tests

After the adsorption equilibrium were achieved at 1 mg/L PhOH and 0.35 m/s flow rate for 24 h, SiO_2_/PDMS coated stirring bar was put into a glass desorption tube containing 250 ul desorption solution (Methanol, Acetone, Acetonitrile, Ethanol) to desorb PhOH under ultrasonic shaking. These samples were also analyzed by HPLC-UV. The stirring bar was put back to methanol under ultrasonic for regeneration.

#### Repeated trials

The adsorption experiments were performed at 1 mg/L PhOH and 0.35 m/s flow rate. After 24 h, the stirring bar was transferred into desorption solution for the desorbing process. The above experiments were repeated 50 times.

### Optimization conditions of HPLC-FTIR

The target contaminants were separated by HPLC and went through the HPLC- FTIR connection component to remove the mobile phase, and then their IR spectra were collected via ATR-FTIR (Technical details are shown in the experimental section of supporting information). The separation and detection performance of HPLC-FTIR technology was evaluated, including mobile phase removal, separation and determination of contaminants and mobile phase (See in supporting information. In addition, 1 ug/L DMP isomers (2, 3-DMP, 2, 4-DMP, 2, 5-DMP, 2, 6-DMP, 3, 4-DMP) were determined by HPLC-FTIR, to highlight the advantage of HPLC-FTIR in the isomer measurements.

### Field water samples analysis

The environmental water samples were collected from the Yellow River, Yangtze River, Liao River, Songhua River, Hai River, and Tai Lake of China. Passive sampling device with SiO_2_/PDMS coated stirring bar was used to enrich target contaminants (Fig. 10). And then SiO_2_/PDMS coated stirring bar was put into methanol solution to desorb phenols including PhOH, 2-CP, 4-CMC, 2, 4-DMP, 2, 4, 6-TCP. Finally, desorption solution was analyzed by HPLC-UV and HPLC-FTIR.

### Instrumentation

A HPLC-UV system (Thermo Fisher Ultimate 3000, USA) with a ternary gradient pump, a 100 ul injection loop, and a variable wavenumber UV detector was used to analyze PhOHs concentration. The separation was performed on a C18 HPLC column (250 mm × 4.6 mm, INERTSUSTAIN C18). The gradient elution included methanol (solvent A), and 10 mmol/L NaH_2_PO_4_ aqueous (solvent B). The gradient elution procedure of PhOHs was as follows: 0–13 min, 57: 43 (solvent A/solvent B, volume fraction), 13.01–20 min, 75: 25 (solvent A/solvent B, volume fraction); and then the ratio of solvent B was decreased to 25 within 2 min and kept for 3 min to equilibrate the column. The flow rate and UV wavenumber were set at 1.0 ml/min, 280 nm, respectively. In addition, the elution procedure of benzaldehyde compounds was 0–10 min, 70:30 (menthol/water, volume fraction) at 0.6 ml/min of flow rate.

FTIR measurements were performed on a Thermo Fisher Nicolet 8700 ATR- FTIR spectrometer equipped with a liquid nitrogen-cooled MCT detector (Hg-Cd-Te semiconductor film, as a photoconduction detector), which improves three more orders of magnitude in sensitivity than a DTGS detector used in the GC-FTIR technology^36, 37^. All spectra were collected at the range of 400–4000 cm^-1^ using a resolution of 4 cm^−1^ and 32 scan times.BET surface area of the prepared catalysts was analyzed using a full automatic analyzer (ASAP2020HD88, Micromeritics Instrument Corp). Field-emission scanning electron microscope (FE-SEM) (S-3000N, Hitachi Limited) was employed to characterize the morphology of particles.

## Conclusions

In this work, SiO_2_/PDMS coated stirring bar was prepared and utilized as passive sampling devices. Compared to commercial PDMS coating, SiO_2_/PDMS coating has many merits including larger surface area, higher adsorption capacity, and higher adsorption rate. After 50 adsorption- desorption cycles, phenol desorption efficiency was more than 75%, indicating that SiO_2_/PDMS coated stirring bar shows good stability and regeneration. In addition, a new method of SiO_2_/PDMS-SBSE-HPLC- FTIR was developed for the determination of phenols isomers in water environment, instead of the total amount of phenols in the previous studies. This new method is very useful to support the performance of water treatment plants as well.

## Electronic supplementary material

Supporting information
