# Supplementary material for: Determination of Phenols Isomers in Water by Novel Nanosilica/Polydimethylsiloxane-Coated Stirring Bar Combined with High Performance Liquid Chromatography-Fourier Transform Infrared Spectroscopy
Source: Sci Rep. 2017 Aug 18;7:8697. doi: 10.1038/s41598-017-09050-2 (PMC5562817; doi:10.1038/s41598-017-09050-2)
Supplement: Supplementary file 1 — Supporting information [file 41598_2017_9050_MOESM1_ESM.doc]

**Determination of Phenols Isomers in Water by Novel Nanosilica/ Polydimethylsiloxane-Coated Stirring Bar Combined with High Performance Liquid Chromatography-Fourier Transform Infrared Spectroscopy**

Bei Zheng,^1^ Wentao Li,^2^ Lin Liu,^3^ Xin Wang,^1^ Chen Chen,^1^ Zhiyong Yu,^1^ and Hongyan Li^*,1^

^1^Key Laboratory of Drinking Water Science and Technology, Chinese Academy of Sciences, Beijing 100085, China

^2^Shenzhen Institute of Information Technology, Shenzhen 518172, China

^3^Northern Engineering Design and Research International Co., Shijiazhuang 050011, China

**HPLC-FTIR connection component**

The HPLC-FTIR connection component was located between the HPLC and FTIR. As shown in Figure S0-1, HPLC-FTIR connection component was composed of an optical platform, ZnSe crystal sample holder, circular driving and rotating platform, heater sleeve, atomizer, cleaning and drying equipment. The operating procedure of the HPLC-FTIR connection component was as follows:


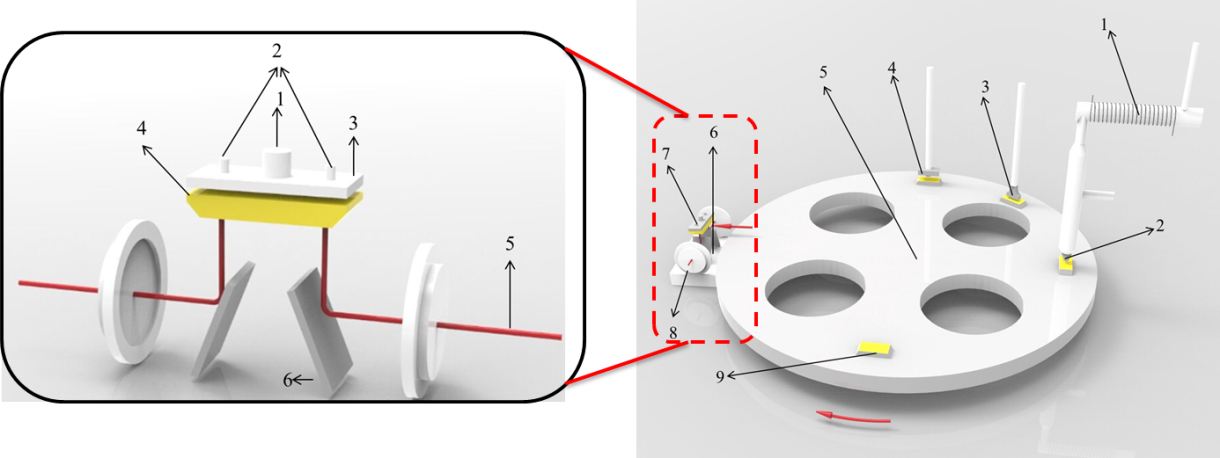


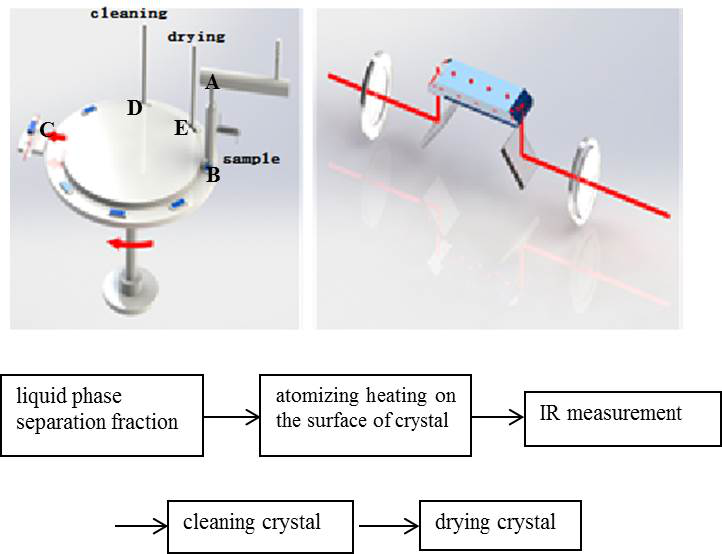


**Figure S0-1.** Coupling HPLC-FTIR interface module components and the flowchart of analysis: left, magnification part (1,injection port;2,vent;3, top cap;4, ZnSe crystal;5, IR light;6,enoscope ); right, main part (1, heater sleeve; 2, atomizer; 3, cleaning equipment; 4, drying equipment; 5, rotating platform; 6, driving device; 7, sample pond; 8, IR light route; 9, ZnSe crystal)

(1) mobile phase removal: target contaminants and mobile phase separated by the chromatographic column passed through the heater sleeve under N_2_, where the mobile phase was removed at different temperatures (273-388K).

(2) sample deposition: a single sample from the atomizer was scattered onto the surface of the ZnSe crystal.

(3) collection of IR spectrum: the ZnSe crystal was moved to the optical platform by a circular driving and rotating platform , where the IR spectrum was collected. In the optical platform, two aluminum mirrors were set to reflect infrared light, which entered into the ZnSe crystal. Multiple reflections of the infrared light in the ZnSe crystal resulted in more contact between the light and the sample on the surface of the ZnSe crystal, producing more spectroscopic information.

(4) crystal cleaning: the ZnSe crystal used was sent to a cleaning unit, where it was washed well by absolute ethyl alcohol drawn by a pump, to avoid any interference with the next measurement.

(5) crystal drying: the clean ZnSe crystal was sent to a drying unit, where it was blown dry at high temperature.

The above operation was controlled with a circular driving and rotating platform, to realize on-line monitoring of the batch samples.

**Optimization conditions of HPLC-FTIR analysis**

Single solutions: 0.1 mg/ml PhOH, 2-CP, 4-CMC, 2, 4-DMP, 2, 4, 6-TCP were dissolved in methanol as single stock solutions, respectively. Mixed solution: 0.1mg/ml of PhOH, 2-CP, 4-CMC, 2, 4-DMP, 2, 4, 6-TCP were dissolved in methanol as a mixed stock solution. In the experiments, the solutions were diluted to 0.1 mg/L. All the stock solutions were stored at 269 K.

In the experiments, the target contaminants were separated through HPLC and entered into the HPLC-FTIR connection component to remove the mobile phase, and then their IR spectra were collected via ATR-FTIR. The correlation coefficient was calculated between the obtained IR spectrum and standard IR spectrum, which was used to evaluate the separation and determination performance of the HPLC-FTIR connection component in the individual contaminant analysis ^1^. Methods have been reported to calculate the similarity of spectra, such as related coefficient, angle cosine, and distance coefficient ^2^. The related coefficient method was used to calculate similarity according to equation (1) ^3,4^:


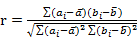
 （1）

In equation (1), a_i_ and b_i_ are the corresponding values for the obtained IR spectrum and standard IR spectrum, respectively, where
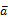
 and
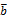
 are average values. The correlation coefficient r was approximately 1.0, indicating that similarity was quite high. In view of the precision and stability of the IR spectra, two spectra were considered to be consistent when similarity was 0.999 or higher. Otherwise, they were considered to be different ^4^. Meanwhile, standard IR spectra of five estrogen compounds were obtained by the KBr pressed disc technique to assure the reliability of the combined technologies.

***Mobile phase removal***

The mobile phase may negative affect the IR measurement ^5^, and heating under N_2_ atmosphere was employed to remove the mobile phase. IR characteristic peaks of mobile phase under different temperatures were shown in table S0. With the increase of temperature from 298K to 388K, the number of IR peaks decreased, and IR peaks disappeared completely at 388 K, which indicates that the interference of mobile phase can be completely removed for IR measurement at 388K. Therefore, the optimum temperature was 388K.

**Table S0 Characteristic peaks of IR spectra of mobile phase at different temperatures**

| Mixture | Characteristic Peaks（cm^-1^） | | | | | | |
| --- | --- | --- | --- | --- | --- | --- | --- |
| 298K mobile | 673.97 | 1029.41 | 1115.68 | 1448.95 | 1646.90 | 2044.90 | 2522.84 |
| phase | 2832.71 | 2944.17 | 3424.41 |  |  |  |  |
| 348 K mobile phase | 699.66 | 1646.69 | 2140.0 | 3424.15 |  |  |  |
| 368 K mobile phase | 699.59 | 1646.84 | 2140.10 | 3424.11 |  |  |  |
| 388 K mobile phase | — | — | — | — | — | — | — |

“—” means undetected

***Separation and determination of contaminants***

To evaluate the separation and determination performance of HPLC-FTIR, correlation coefficient (r) between standard IR spectrum and obtained IR spectrum was calculated^1^. FigureS0-2 shows the correlation coefficients of five phenols (PhOH, 2-CP, 2,4-DMP, 4-CMC, 2,4,6-TCP) at different temperatures. The correlation coefficient was increased to around 1.0 with the increase of temperature till 388K, when IR spectra of five phenols detected completely corresponded to their IR standard spectra. Figure S0-3 shows that IR spectra of five phenols were obtained at different collecting time under the condition of 388K. It could be found that IR spectra were obtained at 7 min, 9 min, 11 min, 13 min, and 20min, while no IR signal was observed for the rest of the collecting time, which was agreed with the results of HPLC-UV (Figure S0-4). The standard IR spectra of five phenols were almost the same as the IR spectra obtained at different collection times, with correlation coefficients of more than 0.999.Therefore, HPLC-FTIR could be used to separate and character the target contaminants.

**Figure S0-2.** Correlation coefficients for five phenols at different temperatures

**Figure S0-3.** IR spectra of five phenols obtained at different acquisition time under 388K

**Figure S0-4.** HPLC of five phenols:PhOH,2-CP,2,4-DMP, 4-CMC, 2,4,6-TCP


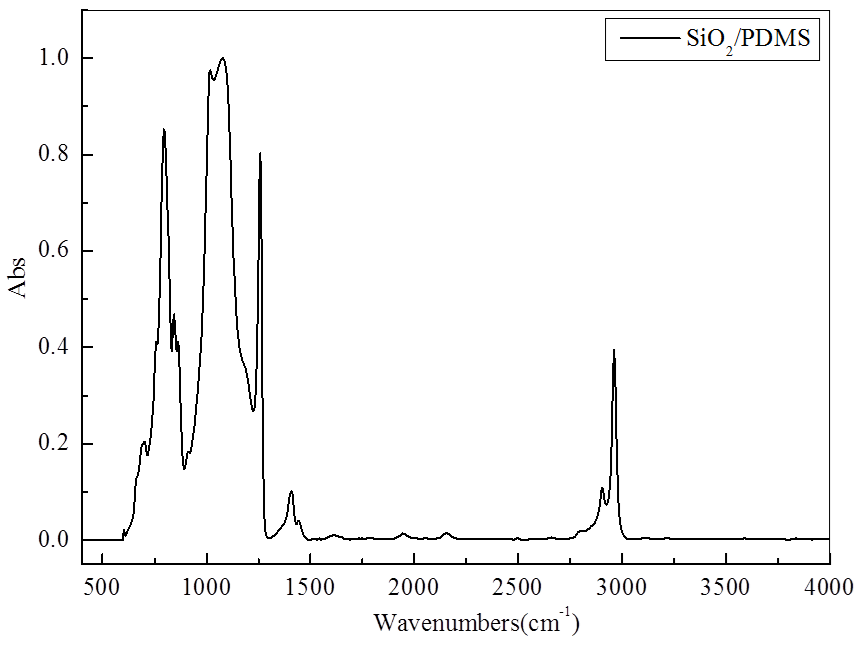


**Figure S1**. FTIR spectra of SiO_2_/PDMS

**Figure S2**. Desorption efficiency of stir bar with different cycling times

**Figure S3.** HPLC chromatogram of DMP isomers: 2,3-DMP;2,4-DMP;2,5-DMP; 2,6-DMP; 3,4-DMP


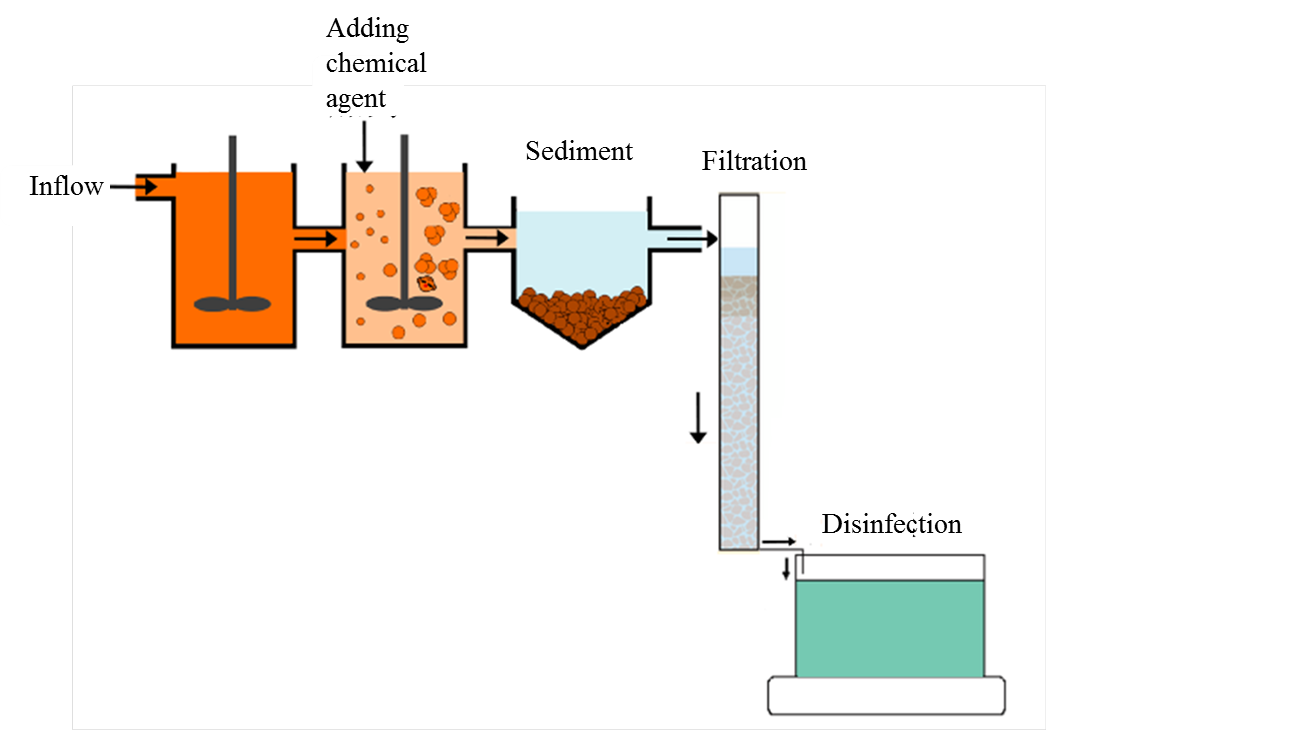


**Figure S4**. Diagram of drinking water treatment process

**Figure S5.** GC-MS of samples and standard Mass spectra of seven isomers


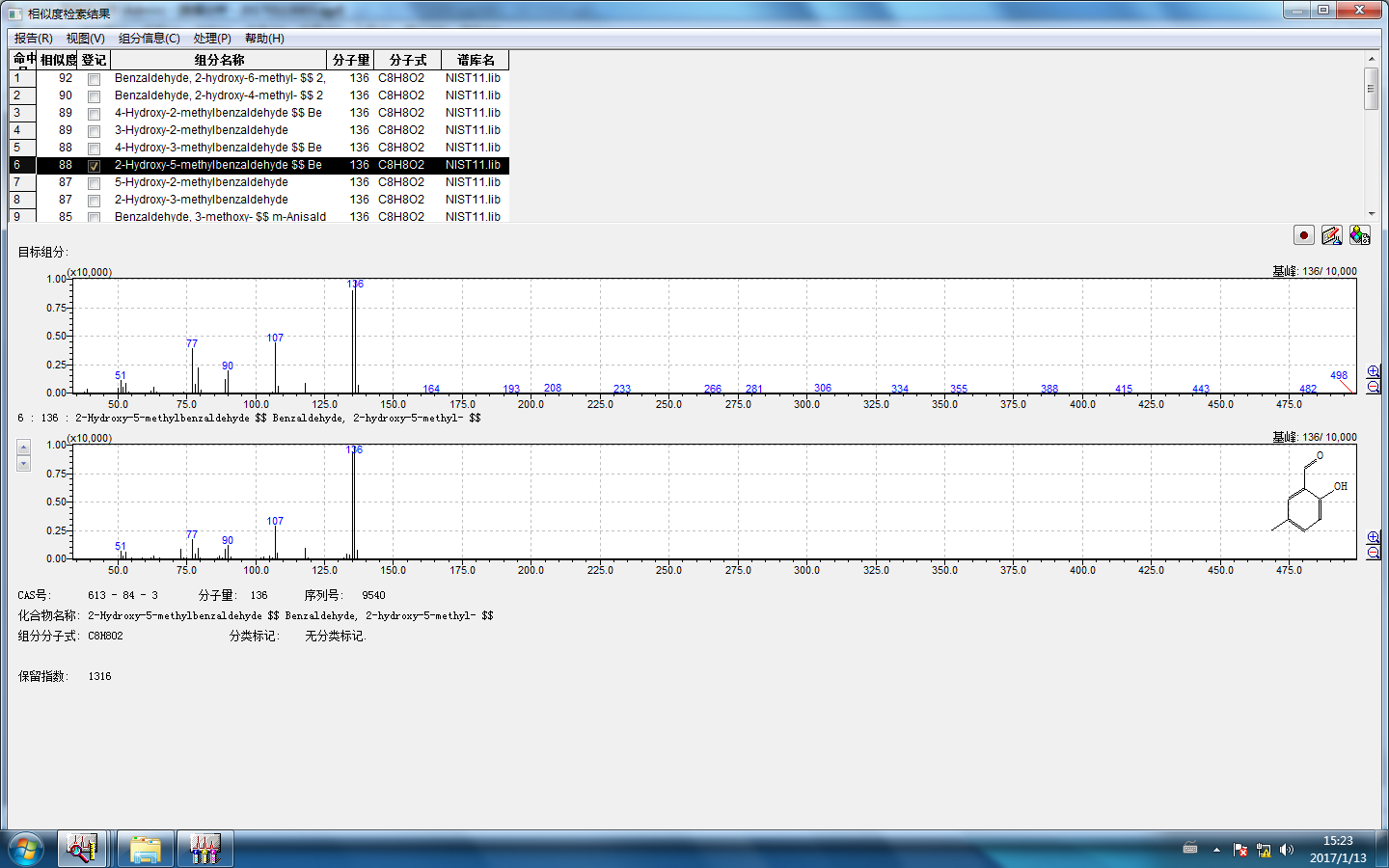

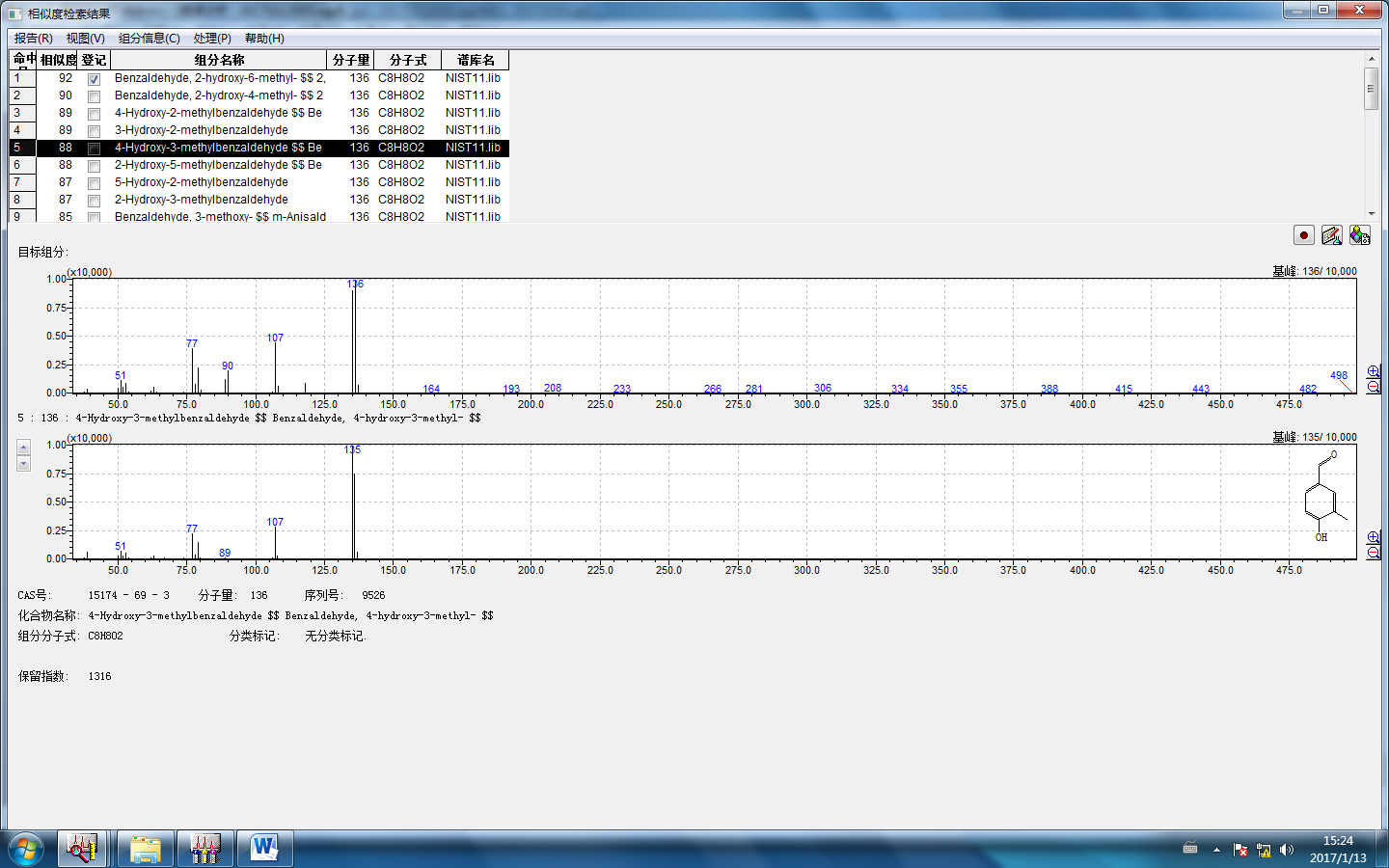

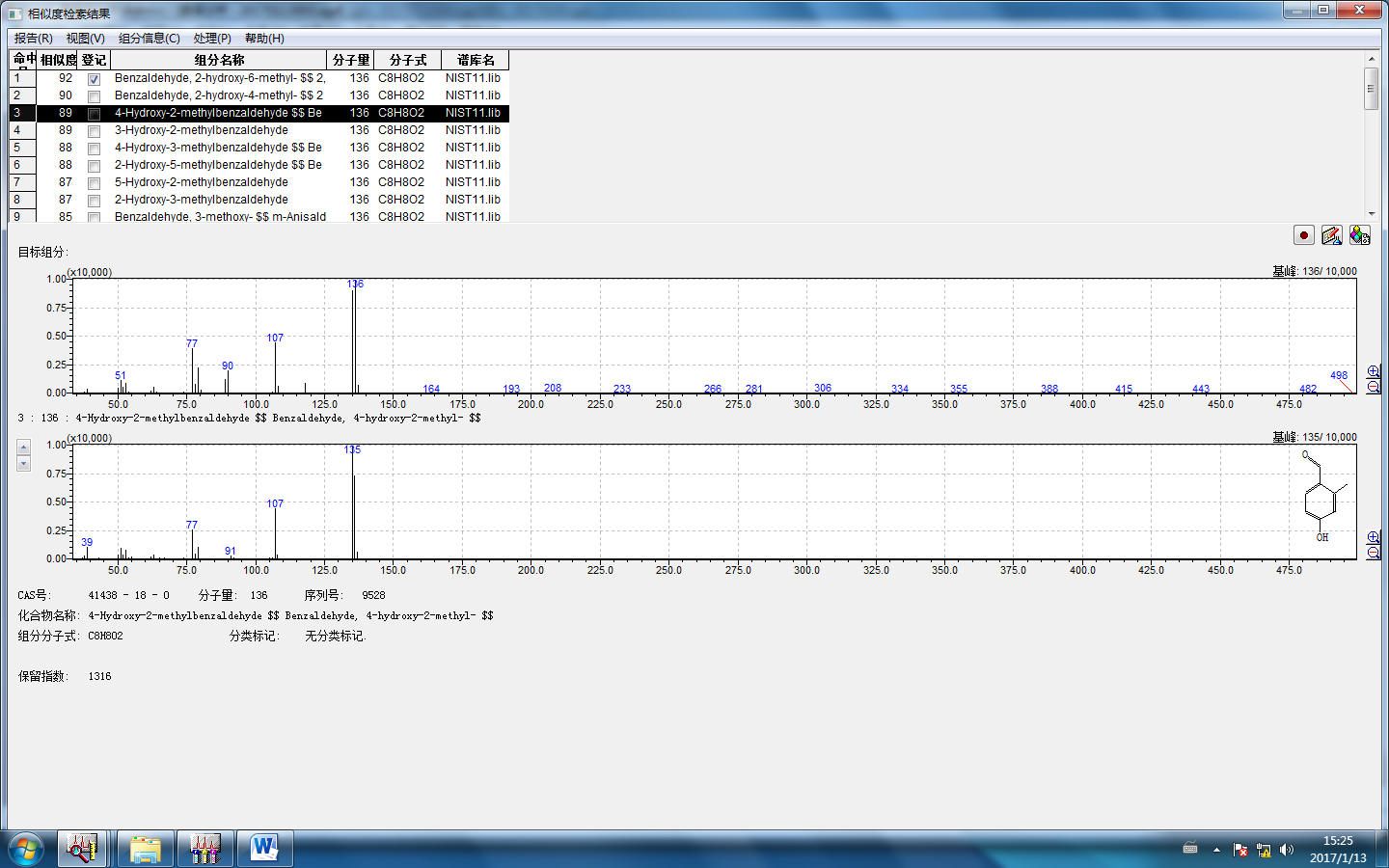

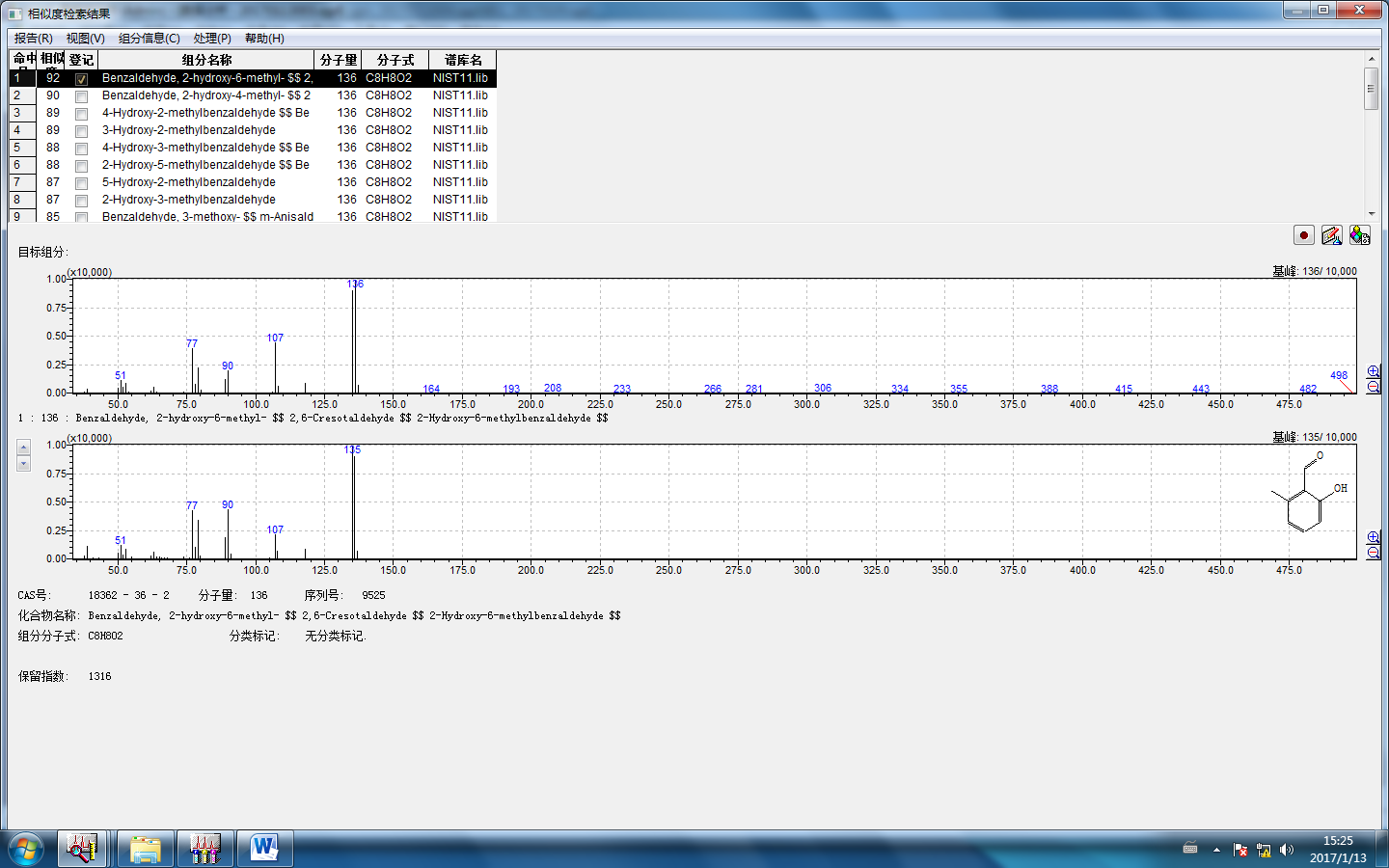

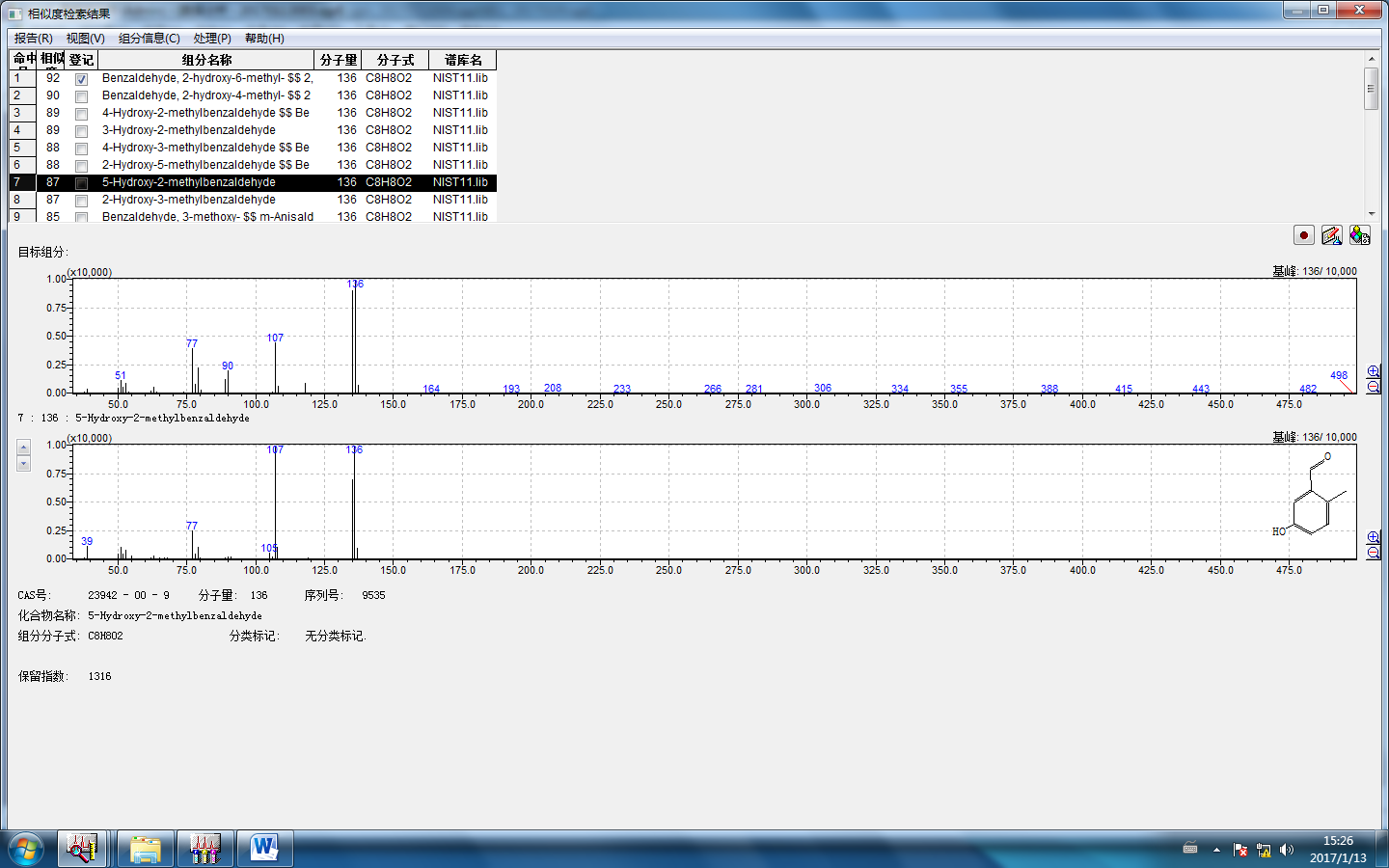

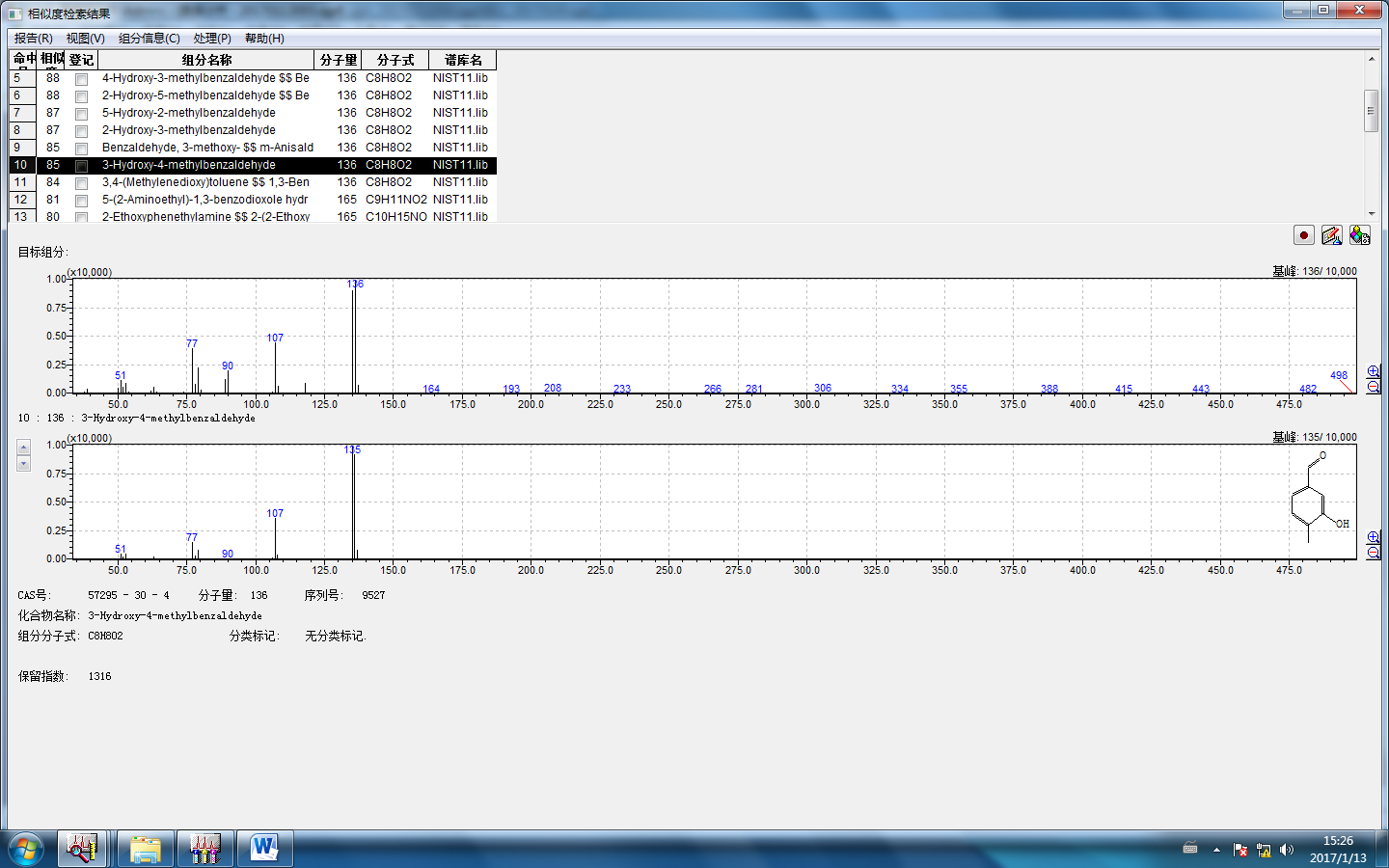

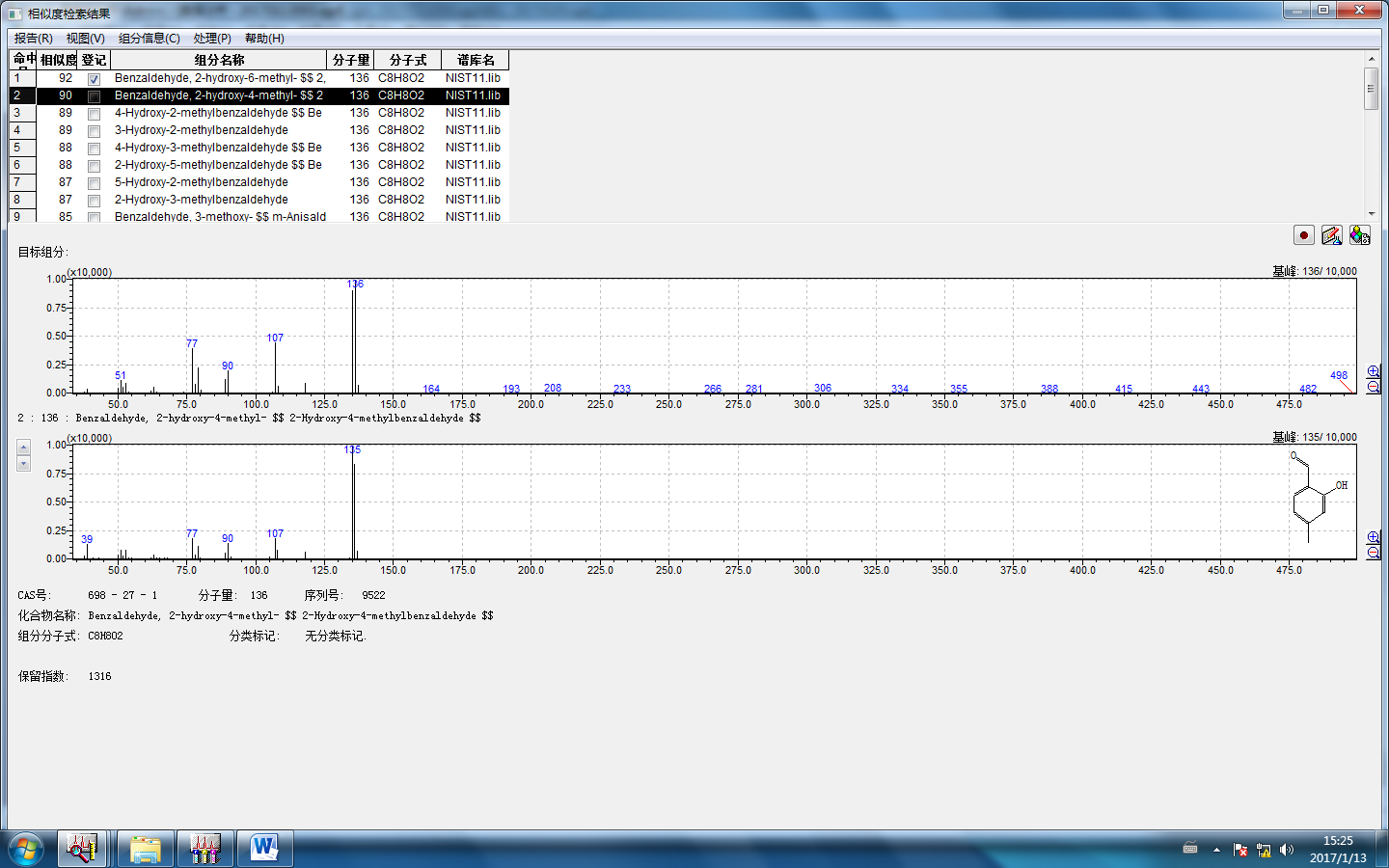


**Figure S6.** FTIR spectra of samples obtained from different treatment processes

**References**

1. Qiao, S. L.,Zhu, Z.Y., Wang, J. J., Chai, Y. F. & Liu, Y. N. *Acad. J .Sec. Mil. Med. Univ.* **25**, 1114-1116(2004).
2. Liu, F., Jiang, Y. H. & He, Y. *Anal. Chim. Acta* **635**, 45-52(2009).
3. Soares, S. F. C. *Anal. Chim. Acta* **689**,22-28(2011)
4. Liu, Y. *Nan Chang Univ.* 18-25(2011).
5. Zheng, B.*et al. Sci. Report* **6**, 32264(2016).
